# Variability and stability of autistic traits in the general population: A systematic comparison between online and in-lab samples

**DOI:** 10.1017/pen.2025.10001

**Published:** 2025-10-01

**Authors:** Qianying Wu, Qianhui Hong, Na Yeon Kim, Ralph Adolphs, Lynn K. Paul, Caroline J. Charpentier

**Affiliations:** 1 Division of the Humanities and Social Sciences, California Institute of Technology, Pasadena, CA, USA; 2 Department of Psychology, University of Maryland, College Park, MD, USA; 3 Brain and Behavior Institute, University of Maryland, College Park, MD, USA

**Keywords:** autism, individual differences, online study, test-retest reliability

## Abstract

The surge of online psychological assessments have brought the autism research community both opportunities and challenges: while they enable rapid large-scale data collection and more power to characterize individual differences, they also bring concerns about data quality, generalizability beyond online samples, and whether autistic traits can be reliably characterized with self-report measures administered online. Here we tackle these concerns by providing a systematic characterization of the autistic traits variability across individuals in a large cross-sectional dataset (*N* = 2826) as well as its temporal reliability within individuals in a test-retest dataset (*N* = 247), with both online and in-lab samples. We measured autistic traits using the Social Responsiveness Scale, 2nd version, Adult Self Report (SRS-2-ASR) – a tool that quantifies individual differences in autistic traits along a continuum for the general adult population. Across individuals, we found elevated SRS scores in online samples and were able to trace this effect to specific subsets of SRS items. SRS scores also covaried with internalizing symptoms, decreased with age, and were lower in women compared to other genders. Within individuals, we find moderate-to-good test-retest reliability of SRS scores over long intervals, with no difference between online and in-lab samples, suggesting robust temporal stability. We conclude that there are systematic differences in autistic traits between online and in-lab samples that are partly explained by systematic population-level differences in internalizing symptoms, particularly social anxiety. Future studies that sample across different populations should measure, control for, or stratify with respect to these factors.

## 1. Introduction

The Social Responsiveness Scale (SRS) is a widely used instrument for measuring the severity of autism spectrum disorder (ASD) through a single summary score (Constantino et al., 2003; Constantino & Gruber, 2012). It was originally developed as a parent- or teacher-rated questionnaire to assess autism severity among children and adolescents (4–18 year olds), and later extended to an adult self report form to quantify social deficits in adulthood (SRS-2-ASR; Constantino et al., 2003; Constantino & Gruber, 2012) and characterize individual differences in autistic traits even in people who do not meet a formal diagnosis of autism. Similar to its original form, the SRS-2-ASR consists of 65 items that are grouped into 5 subscales: social motivation, social awareness, social cognition, social communication, and restricted interests and repetitive behavior. Given its ease of administration (15 min), the SRS-2-ASR has been very extensively used: it has been adapted to various languages and applied to different populations (i.e., clinical and nonclinical samples, different countries); and multiple studies have demonstrated its robust psychometric properties and cross-cultural validity across samples (Chan et al., 2017; Kaplan-Kahn et al., 2021; Nishiyama et al., 2014; Takei et al., 2014). Studies have also reported good sensitivity and specificity of SRS-2 scores to ASD diagnosis (Bölte et al., 2011) as well as moderate to high correlations with scores obtained from gold-standard diagnosis tools such as Autism Diagnostic Observation Schedule (ADOS) and Autism Diagnostic Interview-Revised (ADI-R; Bölte et al., 2011; Kerr-Gaffney et al., 2020; S. Y. Kim et al., 2022). While SRS-2 is not sufficient for ASD diagnosis, it is useful as an easy-to-administer screening tool, especially in adults from the general population.

Traditionally, the SRS has been employed in controlled, lab-based studies, where factors such as participant demographics and testing environments could be tightly regulated. In recent years, online psychological assessments have become increasingly popular as a complementary or even primary approach to lab-based assessments. The development of online crowdsourcing platforms, such as Amazon Mechanical Turk (MTurk), Prolific, etc., have enabled easy access to larger samples that are more diverse and may be historically underrepresented in traditional lab studies (Birnbaum, 2004; Chandler & Shapiro, 2016; Gagné & Franzen, 2023; Palan & Schitter, 2018). Nevertheless, they introduce new challenges. Without a well-monitored environment, participants are more likely to make mistakes or even enter intentionally incorrect answers (Chandler et al., 2020; Jones et al., 2022; Pellicano et al., 2024; Su et al., 2024), which could lead to spurious associations among variables (Huang et al., 2015; Zorowitz et al., 2023). In addition, participants recruited online may differ systematically in demographic, psychological, and psychiatric profiles compared to those who participate in lab-based studies (e.g., local college student samples) (Chandler & Shapiro, 2016; Stewart et al., 2017). Existing studies have reported such differences regarding autistic traits, including higher rate of ASD diagnosis and family history among the general population samples online, as well as higher self-reported autistic traits and different social behavioral performances in diagnosed ASD participants (Mitchell & Locke, 2015; Bazelmans et al., 2024; Banker et al., 2025; Rødgaard et al., 2022).

Along with these growing concerns, a systematic comparison between online and in-lab samples regarding autistic traits measured by the SRS has yet to be conducted. Therefore, in the current study, we aimed to systematically assess and compare self-reported autistic traits in adults across large online samples (*N* = 2332) and lab-based samples (*N* = 494). Using data aggregated across multiple years and studies, we first examined the between-subject variability of the SRS. We compared its total score as well as item-level scores between online and in-lab samples, and identified several other demographic and psychological dimensions that potentially contribute to the SRS heterogeneity in the population.

Next, we reasoned that potential differences in SRS scores between online and in-lab samples could be due to differences in the validity and temporal stability of the measure across study formats. Despite its robust psychometric validity, the test-retest reliability of the SRS-2-ASR has not been fully characterized: to our knowledge, only one study assessed the short-term test-retest reliability of a university sample in Japan over a 2-week interval (Nishiyama et al., 2014). As such, its long-term stability remains unclear, and it may possibly be impacted by assessment settings, which may result in differences between online and in-lab samples. To address this, we employed a smaller test-retest dataset to assess the within-subject stability of the SRS over long-term test-retest intervals (greater than 6 months). We established the test-retest reliability of SRS in the online samples, compared it with that of in-lab samples, and further explored how individual differences in the SRS stability may be associated with other psychological variables.

## 2. Methods

### 2.1. Procedures

We gathered data from various datasets available from previous studies conducted in our labs at the California Institute of Technology, most of which are published (Charpentier et al., 2024; N. Y. Kim et al., 2024; Kliemann et al., 2022; Wu et al., 2024). The main requirements were that these datasets should contain item-by-item SRS scores from the adult self-report version of the SRS (Constantino & Gruber, 2012) in participants from the general population, the date at which SRS was administered, and whether the data was collected as part of an online or in-person (in-lab) study. Through this initial step, we gathered a dataset of *N* = 3746 SRS scores, as well as the following associated variables if they were available: age, gender, sex at birth, race, education, trait anxiety scores (State-Trait Anxiety Inventory - Trait, STAI-T, Spielberger, 1983) depression scores (Beck Depression Inventory-II, BDI-II, Beck et al., 1996), and social anxiety scores (Liebowitz Social Anxiety Scale, LSAS, Liebowitz, 1987). Data from in-lab participants came from Caltech databases such as the Caltech Conte Center (Kliemann et al., 2022) and the more recently established Chen Participant Center. Data from online participants came from five separate studies, four conducted exclusively on Prolific Academic (Prolific, https://www.prolific.com/), and one conducted across both Prolific and Amazon Mechanical Turk (MTurk, https://www.mturk.com/).

*Re-invite study.* To create a test-retest dataset for online participants, we re-invited participants who had completed one of the Prolific studies (first completion during July 2020 to Aug 2021, re-invited during Apr 2024) to complete the following measures again: SRS, STAI-T, BDI-II, and LSAS. Other measures were collected, including Autism Spectrum Quotient (ASQ) scores, state anxiety (STAI-S), NEO five-factor inventory, and questions related to attitudes to the COVID-19 pandemic. However, these measures were ultimately not analyzed given that they were missing or not collected in a large portion of the sample at the first time point. 847 participants were eligible/invited, and *N* = 232 participated. The study was approved by the California Institute of Technology Institutional Review Board (IR22-1199A), and participants were paid a total of $10 for their participation (for a 40 min survey at a rate of $15/h).

### 2.2. Data cleaning and dataset curation

*Initial exclusions.* We excluded participants who had missing data for some SRS items (*N* = 7), which would have resulted in incorrect total SRS scores.

*Cross-sectional dataset.* To build the cross-sectional dataset, aimed at examining variability across individuals, we removed duplicate entries from the same participants. We identified 268 unique participants with two or more entries, i.e. who participated in two or more studies. We kept the earlier data point from the 2020–2024 period of data collection (given that this was when most of the data was collected), resulting in the exclusion of *N* = 311 duplicate entries, and a cross-sectional dataset of *N* = 3428 unique participants (online: 2840 including 1005 from MTurk and 1835 from Prolific, in-lab: 588) before exclusion due to careless responding (see below). Note that while we assumed these participants were unique, we were unable to determine if the same participant could have participated in both an MTurk and a Prolific study, or in both an online and an in-lab study, since we did not have any identifiable information from the online participants. Given the large size of the eligible pool of participants in online platforms (usually greater than 100,000) we reasoned that the probability of duplicate participants across platforms or study formats was extremely low.

*Test-retest datasets.* We built two long-term test-retest datasets, for the in-lab sample and online sample, separately. As the SRS explicitly asked participants to report their behavior over the past 6 months, we considered repeated measures 6 months apart to reflect meaningful changes in autistic traits. To organize the in-lab test-retest dataset, we relied on the duplicate data entries from in-lab participants identified above. From 161 unique in-lab participants with at least two repeats of SRS, we included those who had two measures more than 6 months (183 days) apart. This resulted in an in-lab test-retest dataset of *N* = 55 before careless responding exclusions (see below), and the test-retest intervals ranged from 196 to 2809 days (Figure 2b). To build the online test-retest dataset, we used the data collected during the re-invite study described above (*N* = 232). Participants with missing data (*N* = 4) were excluded, resulting in an online test-retest dataset of *N* = 228 before careless responding exclusions (see below). The test-retest intervals of the online dataset ranged from 947 to 1356 days (Figure 2b). The two test-retest datasets were combined in one, with study format (online or in-lab) as an additional variable. In addition to SRS total, subscales, and item-by-item scores, the dataset contained demographic information, exact dates, and time elapsed between test and retest measures, as well as BDI-II, STAI-T, and LSAS scores at both test and retest.

**Figure 1.**
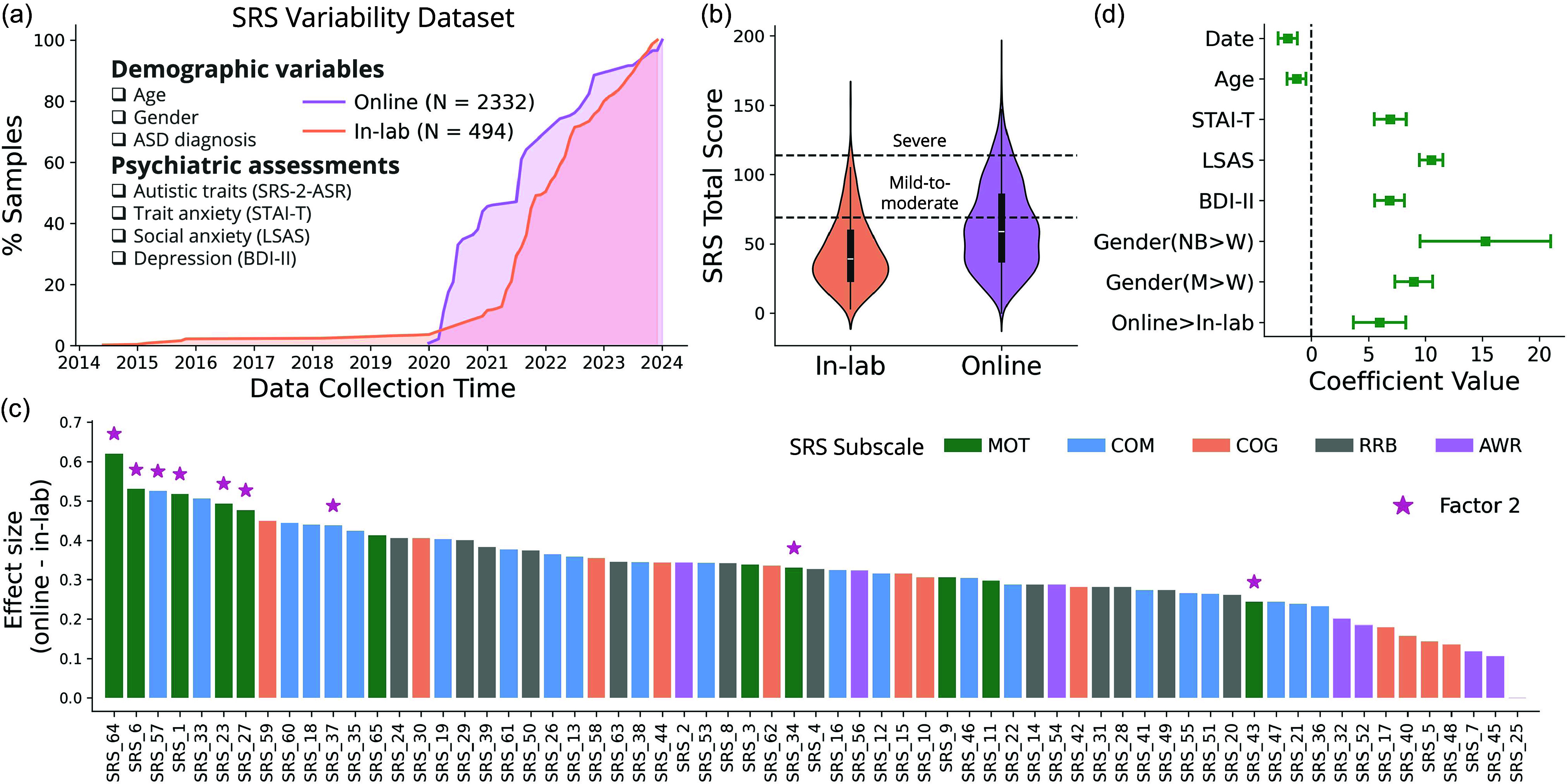
Between-subject variability of SRS. (a) Overview of the dataset. In-lab samples were collected from 2014 to 2024, and online samples were collected from 2020 to 2024. Multiple demographic variables and psychiatric measures were collected. (b) Comparison of the SRS total score between online and in-lab samples. Box plots inside the violin represent the median and interquartile range. The dashed lines represent the typical clinical cutoffs for mild-to-moderate (SRS = 69) and severe (SRS = 114) autism. (c) Difference between online and in-lab samples across all SRS items. Effect size was calculated for each item difference using Cohen’s *d*. Items are colored by their corresponding subscales (MOT: social motivation, COM: social communication, COG: social cognition, RRB: restricted interests and repetitive behavior, AWR: social awareness). Items that belong to the Factor 2 established by (Wu et al., 2024) are highlighted with stars. (d) Regression coefficients when predicting SRS total score. SRS total score was predicted with a multiple linear regression model including the data collection date, participant age, gender, trait anxiety (STAI-T), social anxiety (LSAS), depression (BDI-II) scores, as well as the data collection setting (online vs. in-lab). Regression coefficients and the 95% confidence interval are displayed.

### 2.3. Careless responding

Several analyses were performed on the item-by-item SRS data from the cross-sectional dataset (*N* = 3428) to identify careless responders, who were then excluded from both the cross-sectional and the test-retest datasets. The following metrics were computed with the *careless* package (version 1.2.2) in R (Yentes & Wilhelm, 2021), and participants with above (or below, depending on the metric interpretation) two standard deviations from the mean were excluded, similar to previous studies (Charpentier et al., 2024; Wu et al., 2024):

- Long-string index (to identify straight line responders): excluded *N* = 55 (zscore>2)
- Intra-individual response variability (to identify responders who do not vary their responses in questionnaires with reverse-coded questions): excluded *N* = 128 (zscore<-2)
- Synonyms index (to identify similar responses to synonym items, R > 0.60 was used to determine synonym items): excluded *N* = 83 (zscore<-2)
- Even-odd inconsistency index (to identify inconsistent response between even and odd items of the same subscales): excluded *N* = 414 (zscore>2)

Some participants met the exclusion criteria for more than one index; in total N = 602 unique participants met at least one exclusion criterion, resulting in a final cross-sectional sample size of *N* = 2826. Note that the rate of careless responders was found to be slightly higher in the online sample (*N* = 508 excluded out of 2840, 17.89%) than in the in-lab sample (*N* = 94 excluded out of 588, 15.99%). Of those 602 participants, 36 were also in the test-retest dataset (*N*_excluded_ = 11 in-lab, *N*_excluded_ = 25 online), resulting in a final test-retest sample size of *N* = 247 (*N* = 44 in-lab, *N* = 203 online).

Descriptions of the final datasets are reported in Table 1 (cross-sectional) and 2 (test-retest).

### 2.4. Analyses - between-subject variability

To examine between-subject variability in SRS scores, we used the cross-sectional dataset.

First, we tested whether mean summary SRS scores differed between online (*N* = 2332) and in-lab (*N* = 494) participants by running a Welch’s two-samples *t*-test assuming unequal variances.

Second, we quantitatively examined item-by-item differences between online and in-lab samples, to identify whether overall score differences were likely to be explained by specific items of the SRS. To do this, we calculated the mean score for each SRS item separately for online and in-lab samples, performed two-sample *t*-tests to assess the significance of the difference for each item, followed by False Discovery Rate (FDR) correction for multiple comparisons across the 65 SRS items. We also assessed whether items with the largest difference between study formats were systematically associated with a given SRS validated subscale or factor established in a recent study (Wu et al., 2024).

Third, to provide an integrated explanation of individual variability in SRS scores in our dataset, and characterize how much variance in SRS scores can be explained by other variables, we ran a multiple linear regression predicting SRS from the following variables: format (online vs in-lab), age, gender (Man, Woman, Non-Binary), BDI score, LSAS score, STAI-Trait score, and ordinal date of SRS administration. This regression also allowed examining whether the online vs in-lab difference in SRS scores was robust to controlling for as many variables as possible given our data. Given some missing data in BDI, LSAS, STAI-Trait, gender, and age, the regression was run on a final *N* = 2414 (online: *N* = 1992, in-lab: *N* = 422).

Finally, to better understand the proportion of variance in SRS scores explained by study format as well as by other predictors, we ran several reduced regression models with and without format as a predictor. This allowed us to calculate the partial R^2^ associated with the effect of study format in different contexts, namely (1) the full model (described above), (2) a model where study format is the only predictor of SRS (SRS ∼ format), (3) models missing one of the other predictors (e.g. without social anxiety: SRS ∼ format + age + gender + BDI + STAI-Trait + date, repeated for each predictor), and (4) models without any of the internalizing symptom regressors (SRS ∼ format + age + gender + date). Comparing variance partitioning in (1) vs (2) allowed us to determine how much of the variance explained by study format was unique to study format vs driven by some of the other predictors, while (3) and (4) allowed precisely characterizing which predictor contributed to this difference the most and the specific contributions of internalizing symptoms.

### 2.5. Analyses - within-subject stability

To examine stability in SRS scores, we used the test-retest dataset.

First, we computed the strength of the association between total SRS score at test and at retest, via Pearson’s correlation. We computed the correlation across all participants, as well as separately for in-lab and online samples.

Second, to more robustly assess test-retest reliability *per se*, we computed the intraclass correlation coefficient (ICC) between the initial and repeated measures of SRS. We computed ICC at three levels of granularity (summary scores, each of 5 SRS subscales, and each of 65 SRS items), as well as separately for in-lab and online participants. We applied the two-way random effect ICC model to calculate the absolute agreement of the single measurement score (ICC(2,1)) using the ‘ICC’ function from the *psych* package (version 2.1.9) in R (version 4.1.1). According to a common criterion, ICC < 0.5 indicates poor reliability, 0.5∼0.75 indicates moderate reliability, 0.75∼0.9 indicates good reliability, and ICC > 0.9 indicates excellent reliability (Koo & Li, 2016).

Finally, because the online sample has a much larger sample size than the in-lab sample, we further conducted a bootstrap analysis estimating online sample ICC with subsamples of *n* = 44 that matched the in-lab sample size. We subsampled the online sample by randomly selecting 44 participants with replacement and calculated the ICC of SRS total scores as well as the five subscale scores. This procedure was repeated 1000 times. Based on the 1000 ICC average estimations, we generated the bootstrap distribution, mean, and 95% confidence intervals of the resampled ICC (mean estimation).

### 2.6. Analyses - variability in stability

We examined the SRS stability across individual SRS items, and also across participants. For the former, we assessed whether any specific SRS item exhibited a significant difference between test and retest (with the same FDR-correction procedure described above), as well as whether test-retest item differences varied between in-lab and online samples. We also performed a Pearson’s correlation across the 65 SRS items to examine whether the mean item-by-item differences between in-lab and online samples identified in the variability analyses could be predicted by differences in ICC. In other words, we determined whether items with elevated scores in online compared to in-lab samples also exhibited higher or lower overall test-retest reliability.

To examine individual differences in SRS stability (i.e. the extent to which SRS remains the same between test (T1) and retest (T2)), we ran a multiple linear regression (in *N* = 213 participants given some missing data) predicting the absolute SRS test-retest difference from the following individual difference variables: format (online vs in-lab), sex, age at T1, SRS at T1 (baseline), absolute test-retest difference in BDI, LSAS, and STAI-Trait, the ordinal date, and the test-retest time interval.

## 3. Results

### 3.1. Online samples exhibit elevated SRS scores compared to in-lab samples

To assess variability in SRS in our general population sample, we gathered a cross-sectional dataset of *N* = 2826 unique participants with SRS scores. A summary of the data and variables is presented in Table 1.

**Table 1.** Summary of the SRS variability dataset, broken down by study format

Variable	Online group	In-lab group	Group comparison
% careless exclusion	17.89%	15.99%	
*N* final	2332	494	
Gender (M:W:NB:missing)	1037: 1246: 38: 11	146: 266: 15: 67	*Χ²*(2) = 20.65, *P* < .001
Mean age (SD, *N*_missing_)	34.67 (12.05, *N*_missing_ = 19)	32.12 (11.87, *N*_missing_ = 2)	*T*(722.14) = 4.30, *P* < .001, *d* = 0.21
Mean SRS (SD)	62.58 (30.43)	43.79 (24.62)	*T*(844.29) = 14.729, *P* < .001, *d* = 0.64
Mean SRS-AWR (SD)	7.27 (3.14)	6.04 (2.81)	*T*(776.78) = 8.630, *P* < .001, *d* = 0.398
Mean SRS-COG (SD)	10.08 (5.53)	7.19 (4.64)	*T*(817.30) = 12.093, *P* < .001, *d* = 0.535
Mean SRS-COM (SD)	19.96 (11.89)	13.11 (9.29)	*T*(871.71) = 14.118, *P* < .001, *d* = 0.597
Mean SRS-MOT (SD)	15.05 (6.71)	10.69 (6.04)	*T*(772.73) = 14.254, *P* < .001, *d* = 0.660
Mean SRS-RRB (SD)	10.22 (7.38)	6.75 (5.79)	*T*(867.95) = 11.481, *P* < .001, *d* = 0.487
Mean STAI-trait (SD, *N*_missing_)	44.98 (12.57, *N*_missing_ = 80)	41.65 (11.89, *N*_missing_ = 15)	*T*(723.11) = 5.51, *P* < .001, *d* = 0.27
Mean LSAS (SD, *N*_missing_)	57.61 (29.78, *N*_missing_ = 319)	38.09 (27.12, *N*_missing_ = 25)	*T*(753.51) = 13.76, *P* < .001, *d* = 0.67
Mean BDI (SD, *N*_missing_)	13.80 (11.81, *N*_missing_ = 80)	9.59 (9.68, *N*_missing_ = 15)	*T*(810.42) = 8.30, *P* < .001, *d* = 0.37
Date *N* (<’20:’20-’21:>’21)	0: 1497: 835	17: 227: 250	*Χ²*(2) = 125.75, *P* < .001

SRS subscales: AWR = Social Awareness, COG = Social Cognition, COM = Social Communication, MOT = Social Motivation, RRB = Restricted Interests and Repetitive Behavior. Group comparisons were conducted using the chi-square test (for categorial variables) and Welch’s two-sample *t*-test (for continuous variables), and d denotes Cohen’s *d* effect size.

Our first analysis confirmed our prediction that online participants would exhibit significantly higher SRS scores than in-lab participants (Welch’s two-sample *t*-test, *T*(844.29) = 14.729, *P*<.001, 95% CI = [16.28, 21.29], Cohen’s *d* = 0.637, Fig. 1b, Table 1). This was also true for all five SRS subscales (see Table 1 for details and statistics), including the Restricted Interests and Repetitive Behaviour subscale, which relates more prominently to the nonsocial features of autism, suggesting that the difference is not just driven by elevated social difficulties. We next set out to determine whether this difference could be explained by specific items in the SRS scale, and whether these items may pertain to a specific subscale/factor, either out of the five validated subscales (Constantino et al., 2003; Constantino & Gruber, 2012) or out of the eight factors established in a recent study with online samples (Wu et al., 2024). We found that online participants exhibited higher scores than in-lab participants on all 65 SRS items, and that for all but one item, the difference remained significant after FDR correction for multiple comparisons across the 65 items. To illustrate a few, the three items with the strongest difference between online and in-lab samples were items #64 (“I am much more tense in social settings than when I am by myself.”, Cohen’s *d* = 0.62, p_FDR_ < 0.001), #6 (“I would rather be alone than with others.”, Cohen’s *d* = 0.53, p_FDR_ = 0.003) and #57 (“I tend to isolate myself.”, Cohen’s *d* = 0.525, p_FDR_ = 0.002). Interestingly, the seven items showing the largest difference in effect size (Cohen’s *d*) between online and in-lab participants were all from the motivation (*N* = 5) and communication (*N* = 2) subscales (Fig 1c). These items also largely overlapped with a unique latent factor identified from an earlier study (Wu et al., 2024). In that study, 32 SRS items coming from all 5 subscales belonged to a major factor of SRS (i.e., Factor 1), likely representing an overall mixture of autistic traits; whereas 9 items coming mainly from the social motivation (and social communication) subscales belonged to the Factor 2 that relates to unease during social interactions and preference for isolation. Six out of the top seven elevated SRS items identified here belonged to the Factor 2 of the early study.

**Figure 2.**
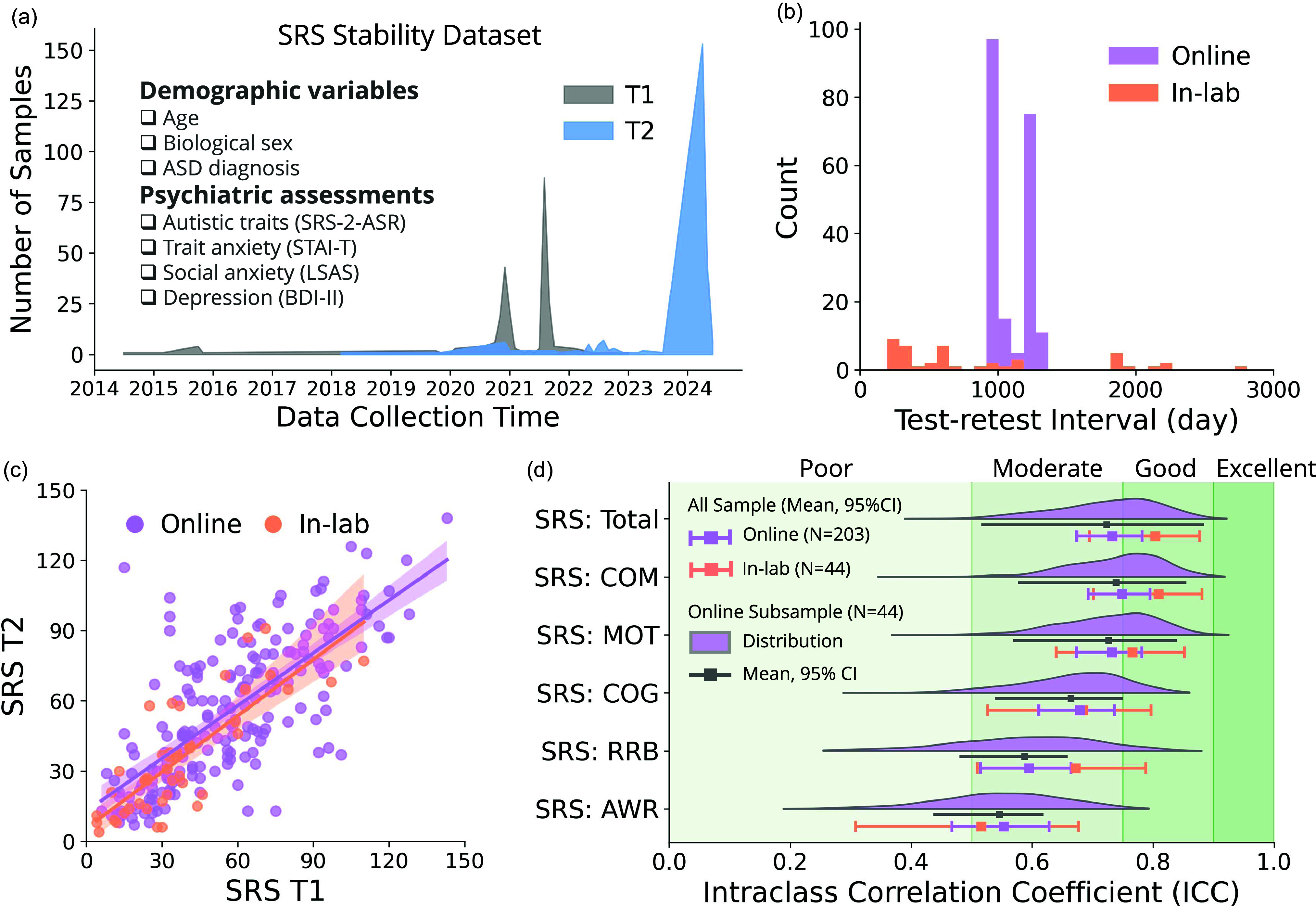
Temporal stability of SRS. (a) Overview of the test-retest dataset. Data of the first (T1) and second (T2) measurement was collected during 2014 to 2024, including multiple demographic variables and psychological assessments. (b) Test-retest interval of the online and in-lab samples, respectively, measured in days elapsed. (c) Correlation between SRS total scores measured at T1 and T2, separately for in-lab and online samples. (d) Test-retest reliability of SRS total score and subscales of both online and in-lab samples, calculated using intraclass correlation coefficient (ICC). For online samples, bootstrapped ICC distributions of subsamples (*N* = 44, matched to the sample size of in-lab sample) were also displayed (half violin plots, points, and errors). ICC < 0.5 indicates poor, 0.5 < ICC < 0.75 indicates moderate, 0.75 < ICC < 0.9 indicates good, and ICC > 0.9 indicates excellent reliability. Error bars represent 95% confidence intervals. COM: social communication subscale, MOT: social motivation subscale, COG: social cognition subscale, RRB: restricted interests and repetitive behaviors subscale, AWR: social awareness subscale.

### 3.2. SRS scores are also associated with age, gender, other internalizing symptoms, and time

We next performed a multiple regression analysis predicting the SRS scores from study format and several additional variables of interest. The aim of this analysis was two-fold: first, to determine whether SRS score differences between online and in-lab samples are robust to controlling for other predictors of SRS, and second, to examine what other factors drive variability in SRS scores in the general population. We noticed that in addition to SRS, scores on other psychiatric scales (BDI, STAI-T, LSAS) were also significantly higher in the online sample compared to the in-lab sample (Table 1). The results showed that when controlling for depression, social anxiety, trait anxiety, gender, age, and date, the effect of study format (online > in-lab) remained significant (*b* = 5.99, T(2405) = 5.13, *P*<.001, 95% CI = [3.70, 8.28]).

All other predictors in the models were found to have significant effects on SRS (Fig. 1d). Specifically, SRS increased with internalizing symptoms, with unique effects of social anxiety (standardized *b* = 10.51, T(2405) = 19.75, *P*<.001, 95% CI = [9.47,11.55], variance explained 13.9%), trait anxiety (standardized *b* = 6.92, T(2405) = 9.66, *P*<.001, 95% CI = [5.51,8.32], variance explained 3.7%), and depression (standardized *b* = 6.86, T(2405) = 10.19, *P*<.001, 95% CI = [5.54,8.18], variance explained 4.1%). SRS scores also decreased with age (standardized *b* = ˗1.30, T(2405) = ˗3.05, *P* = .002, 95% CI = [˗2.14, ˗0.46], variance explained 0.4%), and were higher in men (*b* = 8.96, T(2405) = 10.60, *P*<.001, 95% CI = [7.31,10.62]) and non-binary individuals (*b* = 15.25, T(2405) = 5.22, *P*<.001, 95% CI = [9.52,20.98]) relative to women (variance explained by gender 5.1%). Finally, SRS appeared to decrease over time along the period at which the data was collected (*b* = ˗2.08, T(2405) = -4.79, *P*<.001, 95% CI = [˗2.94, ˗1.23]). In total, these 7 predictors explained 55.1% (total R^2^ = 0.551, adjusted R^2^ = 0.549) of the variance in SRS scores.

We then performed some variance partitioning analyses to further characterize the variance in SRS scores explained by study format, and the extent to which other predictors, particularly internalizing symptoms, may contribute to this difference. In the ‘full’ model containing all predictors, study format was found to uniquely explain 1.1% of the variance in SRS scores, while in a model without any other predictors, study format was found to explain 5.8%, suggesting that a large proportion of the effect of study format on SRS scores can be explained by some of the other predictors. More specifically, we find that 1.9% of this variance is actually shared with social anxiety alone (i.e. in a model without social anxiety only, study format explained 3% of the variance in SRS score instead of 1.1% when social anxiety is included), while 3.8% is shared with all three internalizing symptoms (i.e. in a model without internalizing symptoms, study format explained 4.9% of the variance instead of 1.1%). The above analyses indicate that a large portion of the online vs in-lab difference in SRS scores can be attributed to differences in internalizing symptoms between the two populations, especially social anxiety.

Finally, we explored possible two-way interactions with our variable of interest – study format – to determine whether any of the effects described above might vary between in-lab and online samples. In this regression model, we found that none of the two-way interactions with study format reached significance (all *P*>.12), while the main effects of study format (online>in-lab), LSAS, BDI, STAI-trait, and gender (M > W) remained significant (all *P*<.001). This suggests that the effects of social anxiety, trait anxiety, depression, and gender on SRS do not vary between online and in-lab samples.

Taken together, our results show that the shared variance between SRS, internalizing symptoms and gender, remain similar in the two populations. As such, part of the differences in SRS scores between in-lab and online samples is also shared with and explained by other symptoms.

### 3.3. *SRS scores remain stable across long-term* test-retests*, and across study formats*

With the elevated SRS scores observed in online samples, we asked whether SRS may not be as reliable of a measure in these samples. We also set out to establish the test-retest reliability of SRS scores, subscales and items, over longer timescales (more than 6 months elapsed between test and retest). Our test-retest dataset is summarized in Table 2.

**Table 2.** Summary of the SRS test-retest dataset, broken down by study format

Variable	Online group		In-lab group	
*N* final	203		44	
Mean time elapsed (*T2* - *T1*)	1094 days		876 days	
Sex *N* (M:F:missing)	95: 108: 0		23: 20: 1	
Mean age at *T1* (SD)	34.19 (11.81)		32.02 (8.18)	
*T1* date *N* (<’20:’20:’21:’22:>’22)	0: 69: 134: 0: 0		14: 11: 14: 4: 1	
	*Time 1*	*Time 2*	*Time 1*	*Time 2*
Mean SRS (SD)	56.76 (29.1)	55.46 (29.84)	37.18 (23.48)	35.3 (23.44)
Mean STAI-trait (SD)	44.66 (13.43)	44.18 (14.03)	38.12 (9.61)	37.58 (10.61)
Mean LSAS (SD)	56.75 (31.18)	56.3 (31.51)	26.76 (17.77)	32.47 (25.18)
Mean BDI (SD)	13.15 (12.48)	12.98 (12.84)	6.67 (6.16)	7.1 (6.98)

Note that in the test-retest dataset, we report and use sex rather than gender given that gender had too many missing values in the in-lab sample.

We first found that SRS scores at time 1 and time 2 were highly correlated across the entire sample (*r*(247) = 0.76, *P* < .001), and that there was no mean difference in SRS scores between the two time points (paired *t*-test, *T*(246) = 1.07, *P* = 0.28). More importantly the correlations were also strong and significant when calculated separately for in-lab (*r*(44) = 0.84, *P* < .001) and online (*r*(203) = 0.73, *P* < .001) samples (Fig. 2c). While the correlation in the online group appeared a bit lower numerically, there was no significant difference between the two correlation coefficients (*Z* = 1.004, *P* = 0.315).

To better assess stability, we computed the intra-class correlation (ICC) as an index of test-retest reliability. We found that ICC of the SRS total score for both online and in-lab groups was indicative of moderate to good reliability (online ICC = 0.732, 95% CI = [0.674, 0.782]; in-lab ICC = 0.804, 95% CI = [0.695, 0.877]; Fig. 2d). While in-lab appears to show better reliability, the confidence intervals of the two group ICCs overlapped substantially, indicating that for either the total SRS scores or the SRS subscales, there was no significant difference in test-retest reliability between in-lab and online samples. A bootstrap analysis showed that by matching the sample size of the online sample to the in-lab sample, the uncertainty of the online sample SRS ICC estimation increased, whereas the mean estimation of the online sample ICC remained similar across SRS total scores and subscores (Figure 2d).

### 3.4. Item-wise temporal stability predicts online versus in-lab score differences

**Figure 3.**
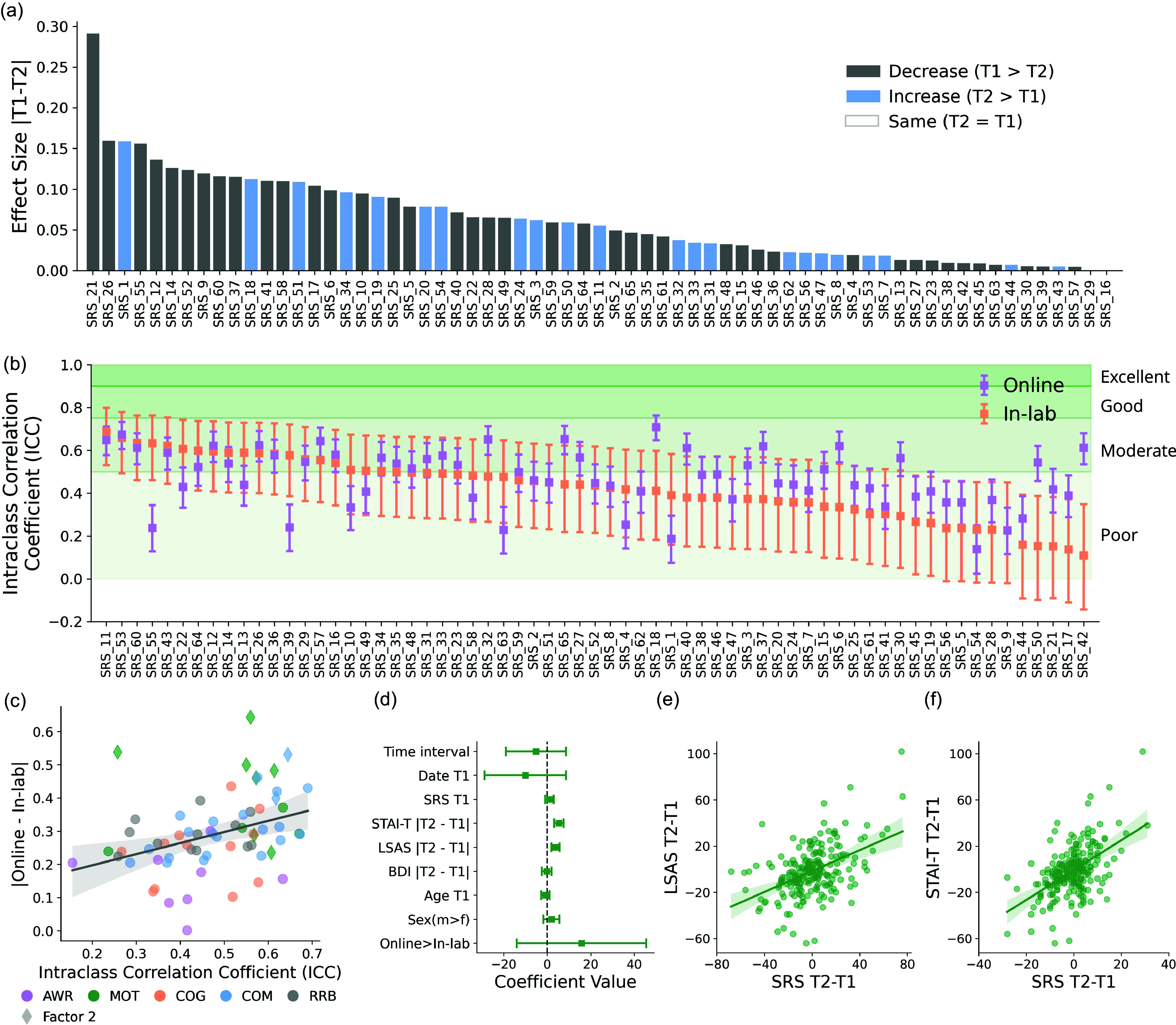
Variability in SRS stability. (a) Effect size of the item-wise SRS score difference between test and re-test. Dark gray bars represent items that have decreased scores over time, blue bars represent items that have increased scores over time. Items are sorted in a descending order of the effect size magnitude. (b) ICC of all the SRS items. Orange indicates in-lab samples, purple indicates online samples. Items are sorted in a descending order of the in-lab ICC. (c) Correlation between item-wise ICC and the difference between online vs. in-lab samples. Items are color coded by the subscales, and items from factor 2 are highlighted. (d) Regression coefficients when predicting within-subject SRS difference. The absolute change in SRS total score between first and second measurements was predicted with a multiple linear regression model including the test-retest interval, first data collection date, SRS score at T1, participant age at T1, sex, study format, and change of trait anxiety (STAI-T), social anxiety (LSAS), and depression (BDI) scores. Regression coefficients and the 95% confidence interval are displayed. (e) Correlation between the signed T2–T1 difference in SRS and that of LSAS. (f) Correlation between the signed T2–T1 difference in SRS and that of STAI-T.

We then explored whether interesting differences might emerge at the item-level in terms of the stability of SRS scores. First, we calculated the mean difference in T2 versus T1 score for each of the 65 SRS items (Fig. 3a). After FDR correction, only one item was found to exhibit a significant difference between T1 and T2 (SRS item #21: “I am able to imitate others’ actions and expressions when it is socially appropriate to do so”). Because this item is reverse coded, the lower score at T2 relative to T1 suggests that the ability to imitate others improved between test and retest. Aside from this item, stability for all other items remained high, with no consistent increase or decrease in item scores between test and retest.

We also examined item-by-item ICC scores. As expected given item-level data, most items exhibited poor test-retest reliability (item-level ICC ranged from 0.11 to 0.71, Fig.3b), though a subset of items appeared to show moderate reliability, both in online and in-lab samples (SRS #11, #53, #60, #43). Focusing on items that exhibited significant test-retest reliability differences between the two groups (confidence intervals not overlapping on Fig. 3b), we found 2 items that were more reliable in in-lab participants than online (#55, #39), while 3 items were more reliable in online participants than in-lab (#18, #50, #42).

More interestingly, we found a significant positive correlation (*r*(65) = 0.36, *P* = 0.003, Fig. 3c) between item-wise test-retest reliability (calculated across all participants rather than separately for each study format) and the item-wise absolute mean score difference between online and in-lab samples (from the large cross-sectional sample; Fig. 1c). This correlation indicates that the more temporally stable a given item is, the more different this item also is between online and in-lab samples.

### 3.5. Trait anxiety and social anxiety contribute to SRS stability, but other factors do not

Our final analysis aimed to assess whether any of the between-subject differences that were found to contribute to the variability in SRS scores, also contribute to their stability between test and retest. To test this, we ran a multiple linear regression predicting the absolute difference in SRS scores between T1 and T2 (“instability” in SRS) from the same instability index in trait anxiety, social anxiety and depression, as well as SRS at T1, study format (online > in-lab), age, sex, date, and time interval between T1 and T2. Because the time interval distributions differed between the two samples (Figure 2a), we first ensured that there was no correlation between the time interval and the change in SRS scores between T1 and T2. We indeed found no correlation in either group (In-lab: *N* = 494, *R* = 0.210, *P* = 0.172; Online: *N* = 2332, *R* = 0.056, *P* = 0.430; Figure S1), suggesting that test-retest reliability comparison between samples in unlikely to be impacted by these interval differences.

The only two significant predictors of SRS instability were instability in trait anxiety (standardized *b* = 7.75, T(203) = 4.89, *P*<.001, 95% CI = [4.62,10.87]) and in social anxiety (standardized *b* = 6.26, T(203) = 5.23, *P*<.001, 95% CI = [3.90,8.62], Fig. 3d). This is consistent with the strong associations between SRS and trait/social anxiety observed in the cross-sectional dataset, further suggesting that within individuals, increases or decreases in anxiety were associated with similar increases or decreases in SRS scores (Fig. 3e–f). Interestingly, the effect of SRS at baseline was not significant, suggesting that participants with high SRS at baseline were not more likely to vary in their SRS scores compared to those with low SRS at baseline. Time intervals also did not predict SRS instability, indicating that at least at these long timescales (> 6 months), stability did not decrease with time elapsed between test and retest. Consistent with earlier results (Fig. 2c–d), study format also had no effect on SRS instability (Fig. 3d). Neither age nor sex had a significant impact on the SRS stability (Fig. 3d).

## 4. Discussion

### 4.1. Online samples exhibit higher autistic traits than in-lab samples

Autistic traits have been viewed as a dimensional construct that characterize difficulties in social cognition, interaction, communication, as well as repetitive or stereotyped behaviors. In addition to a categorical view in clinical settings where individuals are diagnosed either with or without ASD, many studies treat autistic traits as a continuum that is distributed across the entire population. It represents meaningful individual differences about one’s thinking and behavioral patterns especially in the social domain (Landry & Chouinard, 2016), and has been associated with variations in cognitive performance, mental health, and brain functions (Kanne et al., 2009; Kunihira et al., 2006; Mayer, 2017; von dem Hagen et al., 2011; Zhao et al., 2019).

Conducting psychological research through online crowdsourcing platforms has become a new normal, yet whether these findings can generalize to a broader sample is an open question, as there are signs showing potentially different underlying populations between online participants and those traditionally studied in the lab (Chandler & Shapiro, 2016; Stewart et al., 2017). Here, through the use of SRS-2-ASR, we found increased autistic traits in the general population recruited through online platforms. The effect remained significant after controlling for several other individual differences variables, indicating that such outcome is not simply due to different underlying demographic or psychiatric profiles. As the majority of data collection happened during 2020 to 2024, and we found a negative effect of date on the SRS, it is reasonable to speculate that the COVID-19 pandemic and subsequent lockdowns influenced people’s attitudes towards social interactions and distancing, resulting in an increase of SRS scores in the general population in 2020, which then decreased over subsequent years. Nevertheless, since date did not show an interaction effect with format, the potential effect of the pandemic appears not to have contributed to the difference in SRS scores between the online and in-lab population. Altogether, our evidence indicates that there are true differences regarding autistic traits between the populations. The differences might come from a self-selection bias: people who are willing to volunteer for in-person research studies can be more outgoing, comfortable with socialization settings, and exhibit less autistic traits; whereas people who register at online crowdsourcing platforms may have difficulty finding on-site jobs, prefer less in-person social interactions, and show higher autistic traits (Bethlehem, 2010; Difallah et al., 2018).

To further decompose the source of the difference, we examined the online versus in-lab differences in SRS scores for each of the 65 items. Items that had the highest effect sizes shared a common topic – experience of social interaction (e.g., “I am much more tense in social settings than when I am by myself,” “I would rather be alone than with others,” etc.), consistent with a potential self-selection bias. These items largely overlapped with a unique latent factor identified from an earlier study (Wu et al., 2024). Together with the evidence from the current study, those items may be characterizing a specific domain of autistic traits – one’s skill level and preference of being involved in a social situation, such as small talk, group chat, and so on; and participants from online crowdsourcing platforms may specifically experience more difficulties in this domain compared to in-person laboratory volunteers, thus eventually driving an increase in the total SRS scores.

Although our current analyses focused on autistic traits in the general population, the findings speak to the possibility that the diagnosed autistic population recruited online could also exhibit different characteristics from those recruited in-person (Banker et al., 2025; Rødgaard et al., 2022). For example, as online platforms make assessments and research studies more accessible to the broader autism community (i.e. certain subtypes of autistic individuals who were not able to participate in lab-based studies because of their support needs can now perform online tasks at home in a more adapted environment), the ratio of these samples may get higher when a researcher is recruiting from online platforms. Future studies should extend our findings to better understand how different autism subtypes and support levels are represented in research studies and whether this representation varies between online and in-person samples.

### 4.2. Long-term stability of autistic traits measured by SRS

A previous study reported an excellent short-term test-retest reliability of SRS-2-ASR over a two-week interval (Nishiyama et al., 2014), suggesting the questionnaire to be a reliable assessment tool that outputs consistent measures over repeated administrations. Nevertheless, how self-reported autistic traits remain stable during adulthood has not been assessed. Here, we summarized data with test-retest intervals larger than six months (up to eight years) and showed that adult autistic traits measured by SRS have moderate to good long-term reliability (ICC = 0.732 online, 0.804 in-lab), and the inter-individual variations are well-preserved (i.e., high correlation across participants between T1 and T2). In previous work, parent-rated autistic traits in children and adolescents (with and without autism) were found to be reliable over time (Constantino et al., 2009; Holmboe et al., 2014; Robinson et al., 2011; Whitehouse et al., 2011). Our present work extends this finding of test-retest reliability to self-reported autistic traits.

Importantly, the stability of the score was not associated with the test-retest interval, age of testing, sex, study format (online or in-lab), or baseline autistic trait levels. A similar analysis from Constantino et al. (2009) reported the absence of an age effect but the presence of an effect by baseline SRS - higher baseline SRS was correlated with more improvement in autistic symptoms upon the second test. Given that the Constantino et al. (2009) study was conducted on autistic children samples, their finding is likely due to more interventions or support for those who exhibited greater severity (and thus more improvements). Since our samples are the adult general population, such “treatment” effect is unlikely to play a role. Therefore, our findings suggest that autistic traits, at least in the form of a non-clinical, individual difference measure, are stable over time, regardless of the participant demographic profile or the testing environment.

When we further break down the SRS scale to the item level, we observed substantial variations of the test-retest reliability from poor to good levels. By connecting this within-subject stability to the between-subject variability, we found that items that showed more difference between online and in-lab samples also emerged to have better temporal stability. This result consolidates the comparisons between online and in-lab samples, showing that items about experiences of social interactions are robustly higher in the online sample, rather than a consequence of poor measurement reliability. It also illustrates how the temporal stability of a measure is needed to better detect and characterize inter-individual variability.

### 4.3. Autistic traits co-vary with internalizing symptoms

Beside the study format as a key factor, we found that autistic traits were associated with the severity of trait anxiety, social anxiety, and depression, and that a large portion of the differences between online and in-lab SRS scores can be attributed to differences in internalizing symptoms between the two populations. These results are consistent with existing evidence that autistic individuals (or non-autistic individuals with relatively high autistic traits) often report greater internalizing problems (Hallett et al., 2010; Stice & Lavner, 2019). Additionally, here, we also showed that the strength of these associations did not differ between online and in-lab samples. Furthermore, we found that anxiety and social anxiety not only explained part of the between-subject variability in autistic traits but also predicted their within-subject stability: a higher increase in autistic traits correlated with a higher increase in anxiety and social anxiety over time. Note that this association is directional, meaning that instead of a general temporal instability of psychiatric traits (in which case an increase of one trait can be accompanied by a decrease of the other), these mental health symptoms tend to improve or worsen synchronously.

The above findings should remind future researchers to be cautious when interpreting findings about (non-clinical) autistic traits. Given the co-varying patterns among various psychiatric traits, any identified associations between autistic traits and behavioral/physiological/neurological outcomes might be a byproduct of the true underlying relationship with another mental health symptom, and vice versa. To disentangle these effects, one way is to assess the specificity of the findings. For instance, one could control the effect of several other psychiatric measurements when assessing the correlation (or prediction) strength of autistic traits and the target variables. The other way is to replicate the same experiment on clinically diagnosed autistic samples and compare the results with neurotypical participants that are matched on other psychiatric symptom profiles.

On the other hand, given the co-elevation of multiple psychiatric assessment scores observed in the online sample, an alternative explanation is that such pattern reflects domain-general differences between online and in-lab samples, rather than a domain-specific effect of autism (or internalizing symptoms). For instance, lab volunteers might exhibit higher social desirability in self ratings, thus they tend to rate lower on all the psychiatric symptoms (DeVylder & Hilimire, 2015; Kuentzel et al., 2008); such social desirability may be smaller in the online sample as answering questions remotely reduces social pressure. Future studies that systematically compare online and in-lab samples with more diverse dimensions could better distinguish the domain-general or domain-specific nature of the observed differences.

### 4.4. Limitations

Despite the use of a large dataset, we acknowledge the following limitations in the current study.

First, we note that our results are specific to the assessment tool used to measure autistic traits, the SRS-2-ASR, therefore leaving open the possibility that they wouldn’t generalize if another measure – or multiple measures – of autistic traits had been used. Newer self-report assessments, such as the Comprehensive Autistic Trait Inventory (CATI; M. C. English et al., 2025; M. C. W. English et al., 2021) have been designed to cover the autistic phenotype more broadly, and have been validated in large adult samples (both autistic and non-autistic) online. Future studies should investigate whether these other assessment tools, particularly those better at characterizing “non-social” autistic features (e.g. special interests, camouflaging, repetitive behaviors, etc) as well as observational reports by family or caregiver, would differ to the same extent between in-lab and online participants.

Second, since the dataset is aggregated from various sources with each of them having collected different sets of variables, we were not able to retrieve enough sample size for a lot of variables that could have been of interest to the analysis. For example, a major source of variation in demographic profiles could come from participants’ race, ethnicity, level of education, and socioeconomic status. With this information, more thorough analysis would have been possible to uncover whether these demographic variables might further contribute to the autistic trait differences between the online and in-lab populations (beyond internalizing symptoms, age, and gender).

Third, in the test-retest analyses, the test-retest intervals between online and in-lab samples had largely different distributions. As the former was more homogeneous and mainly around 3 years, the latter was more heterogeneous and spread out between 6 months and 8 years, making it difficult to appropriately match the intervals between the two samples. Additionally, the smaller in-lab sample size may have led to a less precise estimation of its test-retest reliability (i.e., larger confidence interval of ICC). Despite our efforts in controlling for differences in test-retest intervals and bootstrapping to match sample sizes, one should interpret the stability comparisons between the online and in-lab samples with caution. Note however that even with this difference in test-retest intervals, we still find comparable test-retest reliability values in the two samples, reducing the potential concern due to this limitation. Still, future studies should continue examining the stability of autistic traits in different types of samples with more balanced designs.

Finally, each individual dataset may have been biased by eligibility criteria and screening procedures specific to each respective study, which we were not able to control for; so while the data was from the general population, the sample may not have been fully representative of the general population, in particular when it comes to diagnosis or history of psychiatric disorders. Given our findings related to internalizing symptoms, future work is needed to assess how different clinical diagnoses such as social anxiety disorder or major depression may be over- or under-represented in online vs in-lab samples, in the general population and in ASD specifically.

## 5. Conclusion

In this study, we characterized the between-subject variability and within-subject temporal stability of autistic traits in the general adult population, measured by a well-established self-report scale, SRS-2-ASR. We found higher autistic traits in online samples compared to lab-based samples, with the strongest differences mainly stemming from items reflecting greater challenges during social interactions among the online participants. The long-term test-retest reliability of the SRS-2-ASR was moderate-to-good for both online and in-lab samples. Both the variability and stability of autistic traits can be largely explained by the severity and temporal fluctuations of internalizing symptoms, including anxiety, social anxiety, and depression. Future studies should extend the current findings to the diagnosed autistic population and better understand how the trait profiles could vary across different sources of clinical samples.

## Supporting information

Wu et al. supplementary material Wu et al. supplementary material

## Data Availability

De-identified data and code are available at: https://github.com/wuqy052/SRS_variability_stability

## Supplementary material

To view supplementary material for this article, please visit https://doi.org/10.1017/pen.2025.10001.

## Social media summary

Self-reported autistic traits are higher in online than in-lab samples, but remain stable over time in both groups.
