# Supplementary material for: Variability and stability of autistic traits in the general population: A systematic comparison between online and in-lab samples
Source: Personal Neurosci. 2025 Oct 1;8:e5. doi: 10.1017/pen.2025.10001 (PMC12516608; doi:10.1017/pen.2025.10001)
Supplement: Wu et al. supplementary material [file S2513988625100011sup001.docx]

# Supplementary figures

**Figure S1.** Association between SRS score differences and the test-retest interval. Changes in the SRS scores between the first and second measurement (calculated as the absolute difference) were not associated with the test-retest interval for both (a) in-lab and (b) online data.
